# Transdiagnostic brain correlates of self-reported trait impulsivity: A dimensional structure-symptom investigation

**DOI:** 10.1016/j.nicl.2023.103423

**Published:** 2023-04-28

**Authors:** Anna-Chiara Schaub, Marc Vogel, Undine E. Lang, Stefan Kaiser, Marc Walter, Marcus Herdener, Johannes Wrege, Matthias Kirschner, André Schmidt

**Affiliations:** a University of Basel, Department of Psychiatry (UPK), Basel, Switzerland; b Department of Psychiatry, Psychotherapy and Psychosomatics, Psychiatric Hospital, University of Zurich, Switzerland; c Division of Adult Psychiatry, Department of Psychiatry, Geneva University Hospitals, Switzerland

**Keywords:** Impulsivity, Anhedonia, Transdiagnostic, Inferior frontal gyrus, Putamen

## Abstract

Impulsivity transcends psychiatric diagnoses and is often related to anhedonia. This ad hoc cross-sectional investigation explored 1) whether self-reported trait impulsivity mapped onto a common structural brain substrate across healthy controls (HCs) and psychiatric patients, and 2) in a more exploratory fashion, whether impulsivity and anhedonia were related to each other and shared overlapping brain correlates. Structural magnetic resonance imaging (sMRI) datasets from 234 participants including HCs (n = 109) and patients with opioid use disorder (OUD, n = 22), cocaine use disorder (CUD, n = 43), borderline personality disorder (BPD, n = 45) and schizophrenia (SZ, n = 15) were included. Trait impulsivity was measured with the Barratt Impulsiveness Scale (BIS-11) and anhedonia with a subscore of the Beck Depression Inventory (BDI). BIS-11 global score data were available for the entire sample, while data on the BIS-11 2nd order factors attentional, motor and non-planning were additionally in hand for a subsample consisting of HCs, OUD and BPD patients (n = 116). Voxel-based morphometry analyses were conducted for identifying dimensional associations between grey matter volume and impulsivity/anhedonia. Partial correlations were further performed to exploratory test the relationships between impulsivity and anhedonia and their corresponding volumetric brain substrates. Volume of the left opercular part of the inferior frontal gyrus (IFG) was negatively related to global impulsivity across the entire sample and specifically to motor impulsivity in the subsample of HCs, OUD and BPD patients. Across patients anhedonia expression was negatively correlated with left putamen volume. Although there was no relationship between global impulsivity and anhedonia across all patients, only across OUD and BPD patients anhedonia was positively associated with attentional impulsivity. Finally, also across OUD and BPD patients, motor impulsivity associated left IFG volume was positively linked with anhedonia-associated volume in the left putamen. Our findings suggest a critical role of left IFG volume in self-reported global impulsivity across healthy participants and patients with substance use disorder, BPD and SZ. Preliminary findings in OUD and BPD patients further suggests associations between impulsivity and anhedonia that are related to grey matter reductions in the left IFG and putamen.

## Highlights

• Self-reported trait impulsivity is related to decreased volume of the left inferior frontal gyrus (IFG)
• Dimensional relationship evident across healthy participants and diverse psychiatric patients.
• Association between impulsivity and anhedonia.
• Volume decreases in IFG and putamen may underlie impulsivity-anhedonia associations.

## 1 Introduction

Determining whether basic dimensions of functioning across traditional psychiatric boundaries map onto common neural substrates may facilitate the detection of novel targets for developing biomarker-based transdiagnostic treatments (Insel et al., 2010, Insel, 2014). The Research Domain Criteria (RDoC) (Cuthbert and Insel, 2013) incorporate a dimensional approach to psychopathology by linking neuroscientific mechanisms to full range of variation (from normal to abnormal) in basic neurobehavioral functioning. Within the RDoC framework the cognitive control system is considered as one core domain (Cuthbert and Insel, 2013) and a breakdown in this system is thought to underlie impulsivity (Bari and Robbins, 2013, Dalley et al., 2011), a personality trait that transcends across categorical diagnoses such as for instance personality disorders, substance use disorders (SUD) or schizophrenia (SZ) (for a review see Robbins et al., 2012). Impulsivity is a multifaceted construct comprising on one hand rapid-response impulsivity (also referred as response inhibition or stopping impulsivity) and reward-delay impulsivity on the other (i.e. delay discounting or waiting impulsivity), with dissociable underlying cortico-striatal circuits (Dalley et al., 2011). Derived from evidence of task-based fMRI studies, waiting impulsivity thereby depends upon interactions between the dorsal and ventral anterior cingulate cortex (ACC), ventromedial prefrontal cortex (PFC), hippocampus, amygdala and structures in the ventral striatum (Dalley et al., 2011, Dalley and Robbins, 2017), while stopping impulsivity mainly involves interactions between the dorsal striatum (caudate-putamen), motor cortex, ACC, and right inferior frontal gyrus (IFG) and orbitofrontal cortex (OFC) (Dalley et al., 2011, Dalley and Robbins, 2017). However, it of note that the left IFG also seems critical for suppressing prepotent but inappropriate responses (Goya-Maldonado et al., 2010, McDonald et al., 2017, Swick et al., 2008, Swick and Chatham, 2014, Wager et al., 2005).

An often used self-report to assess trait impulsivity is the Barratt Impulsiveness Scale (BIS-11) (Patton et al., 1995). The BIS-11 comprises three subtraits of impulsivity, namely non-planning impulsivity (orientation to the present rather than to the future), attentional (a lack of focus on the ongoing task) and motor impulsivity (acting without thinking). However, it remains unknown whether self-reported impulsivity (i.e. BIS-11) map onto the fMRI-derived cortico-striatal circuits of waiting and stopping impulsivity across psychiatric diagnoses. In healthy controls (HCs), negative correlations between left OFC and ACC grey matter volume and BIS-11 total scores have been reported and between right / left OFC volume and BIS-11 non-planning and motor scores, respectively (Matsuo et al., 2009). Similarly, Schilling et al found negative correlations between cortical thickness of the left middle frontal gyrus and the BIS-11 total, motor and non-planning scores (Schilling et al., 2012). In individuals with cocaine use disorder (CUD), grey matter volume of the left dorsolateral and ventrolateral PFC correlated negatively with BIS-11 attention and non-planning (Meade et al., 2020), while in another study with cocaine users BIS-11 attentional and motor impulsivity were negatively related to volume of the right OFC and superior frontal gyrus, respectively (Crunelle et al., 2014). BIS-11 total scores in patients with opioid use disorder (OUD) were negatively correlated with volumes in the bilateral medial PFC and dorsolateral PFC (Qiu et al., 2013). Harmful drinking has been associated with volume reduction in the left IFG, which was correlated with increased BIS-11 attentional impulsivity (Gröpper et al., 2016). In patients with borderline personality disorder (BPD), bilateral dorsolateral PFC grey matter was inversely associated with the BIS-11 total score, and attention and motor subscale (Sala et al., 2011). In patients with schizoaffective disorder, BIS-11 total scores were significantly associated with left lateral OFC volume, which was also significantly associated with BIS-11 motor and non-planning scores (Nanda et al., 2016).

Impulsive actions often take place in the context of negative emotions, such as anhedonia and apathy, which may drive the development of impulsive acts as a form of “self-treatment” to diminish negative emotional states (Blum et al., 2000, Houeto et al., 2016). Anhedonia also cuts across traditional disease boundaries (Whitton et al., 2015) and is highly prevalent in major depressive disorder (MDD) (Treadway and Zald, 2011), SZ (Horan et al., 2006), SUD (Garfield et al., 2014), and BPD (Marissen et al., 2012), to mention only a few. In a recent network analysis in healthy participants, anhedonia was connected to BIS-11 non-planning and attention (Zhang et al., 2022). Anhedonia was also found to be positively related to dysfunctional impulsivity (acting with less forethought) in BPD patients (Marissen et al., 2012). In patients with bipolar disorder, anhedonia was positively correlated with BIS-11 attention (Swann et al., 2008). Anhedonia scores were further positively related to BIS-11 total scores in SZ patients, but negatively related in MDD patients (Amr and Volpe, 2013). Anhedonia subsumes a consummatory (‘liking’) and anticipatory (‘wanting’) aspect with dissociable front-striatal circuits: whereas the ventral striatum thereby may rather mediate the consummatory aspect (Peciña and Berridge, 2005), a wider network consisting of the ventral striatum but also other neural structures such as the caudate, pallidus and putamen are involved in anticipatory anhedonia (Berridge, 1996, Schultz et al., 2000). In a previous study, we found that anhedonia severity was negatively related to the left putamen volume across patients with MDD, SZ, BPD, OUD, and CUD, which we interpreted as brain substrate for low anticipatory pleasure and psychomotor retardation (Schaub et al., 2021). Notably, reduced left putamen myelination and volume has also been associated with impaired impulsivity in healthy individuals (Nord et al., 2019) and neuropsychiatric disorders (Luo et al., 2019).

The main aim of this study was to explore whether there is a dimensional relationship between grey matter volume and global impulsivity or its subdomains attention, motor and non-planning across HCs, patients with SZ, BPD, OUD and CUD. We were particularly interested to test whether transdiagnostic impulsivity expression may rather map on a prefronto- dorsostriatal (stopping impulsivity) or ventrostriatal network (waiting impulsivity). We predicted negative relationships between BIS-11 total and motor scores and grey matter volume in the PFC regions underlying stopping impulsivity such as the IFG and OFC, and between BIS-11 non-planning scores and PFC regions of waiting impulsivity such as the ventral ACC and ventromedial PFC. The second more exploratory aim was to investigate relationships between impulsivity (BIS-11 scores) and anhedonia scores and their corresponding brain correlates. At the behavioral level, we hypothesized a positive correlation between anhedonia and BIS-11 non-planning and attentional impulsivity across patients. In our previous study with the same transdiagnostic sample including patients with depression and first-episode psychosis, we found a negative relationship between anhedonia and putamen volume across patients (Schaub et al., 2021). We also expected to see this negative putamen – anhedonia relationship in the present investigation with SZ, BPD, OUD and CUD patients (depressed and first-episode psychosis patients removed) and that the anhedonia-related putamen volume would be associated with the brain correlate of BIS-11 attention and non-planning impulsivity.

## 2 Materials and methods

This work includes a subsample of a previous analysis decoding neural correlates of anhedonia across psychiatric diagnoses, which also included depressed and first-episode psychosis patients (Schaub et al., 2021). Only individuals with available BIS-11 data were included in this follow-up analysis.

### 2.1 Participants

Two hundred thirty-four participants were included in this reanalysis. Samples from two different centres were included: HCs (n = 49), outpatients with OUD (n = 22) and BPD (n = 45) were recruited by clinicians from the Department of Psychiatry (Universitäre Psychiatrische Kliniken, UPK), University of Basel, Switzerland, and a sample of HCs (n = 60), patients with CUD (n = 43) and chronic SZ (n = 15) were recruited from the Department of Psychiatry, Psychotherapy and Psychosomatics, University of Zurich, Switzerland. All participants provided written informed consent, and the studies were approved by the local ethics committees.

Patients were diagnosed with the German Version of the Structured Clinical Interview for DSM-5 (SCID I and II (Wittchen et al., 1997)). Except for nicotine dependence, all patients were without current neurological or severe medical disorders and history of head injury, and were above 18 and below 65 years old. Disorder-specific analyses in OUD (Schaub et al., 2022, Schmidt et al., 2021), BPD (Wrege et al., 2019), CUD (Engeli et al., 2021, Kirschner et al., 2018) and SZ (Stepien et al., 2018) have previously been published. Here, we present an ad-hoc analysis with combined samples.

HCs (total n = 109) were recruited by advertisement and screened for any neuropsychiatric disorder using the M.I.N.I. (Lecrubier et al., 1997) to ensure that they had no previous or present psychiatric illness. The M.I.N.I is a diagnostic structured interview compatible with DSM-III-R and ICD-10 criteria that takes half as long as administration of corresponding sections of the SCID (Lecrubier et al., 1997), and such well suited for the screening of a healthy control group. All control participants were required to have no personal lifetime psychiatric disorder and no family history of any psychiatric disorder, head trauma, neurological illness, serious medical or surgical illness, or substance abuse. All participants were further screened to exclude insufficient German language fluency. A detailed description of the study sample including medication is summarized in Table 1.

**Table 1:** Sociodemographic and clinical characteristics of study participants.

	HC (n = 109)	OUD^a^ (n = 22)	BPD^b^ (n = 45)	CUD^c^ (n = 43)	SZ^d^ (n = 15)	Between-group statistics
Sex; female/male	56/53	6/16	35/10	13/30	2/13	χ^2^ = 32.94, p < 0.001
Age in years, mean (SD)	30.07 (7.18)	50.77 (5.84)	27.51 (8.03)	30.53 (7.18)	32.33 (9.44)	F(4, 233) = 34.40, p < 0.001; HC < OUD, p < 0.001; CUD < OUD, p < 0.001; SZ < OUD, p < 0.001
Education in years, mean (SD)	13.76 (0.32)	10.00 (1.11)	13.01 (2.47)	11.44 (3.24)	11.90 (1.85)	F(4, 233) = 8.33, p < 0.001; HC > CUD, p < 0.001; HC > OUD, p < 0.001;
Smoking, cigarettes per day, mean (SD)	5.44 (7.05)	17.32 (8.32)	11.93 (10.72)	11.63 (10.09)	20.60 (24.12)	F(4, 233) = 12.75, p < 0.001 CUD > HC, p < 0.009; OUD > HC, p < 0.001; SZ > HC, p < 0.001; BPD > HC, p < 0.004
BDI anhedonia score, mean (SD)	NA	3.77 (2.49)	4.64 (2.83)	1.72 (1.76)	1.87 (1.30)	F(3, 124) = 13.994, p < 0.001 OUD > CUD, p = 0.005; BPD > CUD, p < 0.001; BPD > SZ, p < 0.001

OUD, opioid use disorder; BPD, borderline personality disorder; CUD, cocaine use disorder; SZ, schizophrenia; SD, standard deviation; BDI, Beck Depression Inventory; NA, Not applicable.

a Patients with OUD were actively enrolled in a heroin-assisted therapy for at least 6 months (mean (SD) 7.295 ± 4.74 years) with an unchanged dose of diacetylmorphine during the previous 3 months (mean (SD) dose: 341.82 ± 126.52 mg). Duration of opioid use was 21.82 ± 5.82 years with an age of onset of 19.09 ± 3.41 years.

b 23 BPD patients were medication-free. 20 BPD patients were treated with antidepressants (mean (SD) fluoxetine equivalence dose: 44.00 ± 31.90 mg), of whom 6 were additionally treated with antipsychotics (mean (SD) chlorpromazine equivalence dose: 188.29 ± 190.67 mg) and 3 with antiepileptics (mean (SD) dose: 350 ± 132.29 mg). 2 patients exclusively received antipsychotics (mean (SD) chlorpromazine equivalence dose: 159.75 ± 175.72 mg).

c CUD patients were not medicated.

d 14 (out of 15) SZ patients were treated with antipsychotics: 4 × clozapine (1x50mg, 1x75mg, 1x175mg, 1x200mg), 3 × aripiprazole (1x5mg, 1x10mg, 1x15mg), 1 × 80 mg lurasidone, 3 × olanzapine (2x15mg, 1x20mg), 2 × paliperidone (1x100mg, 1x150mg), 1 × 200 mg quetiapine.

### 2.2 Assessment of impulsivity and anhedonia

The BIS-11 (Patton et al., 1995) was used to assess impulsivity. The BIS-11 total score was available for the entire sample (n = 234). In the Basel sample (n = 116), also each of the 30 BIS-11 items were in hand, enabling additional subanalyses with the 2nd order factors ‘attentional’, ‘motor’ and ‘non-planning’ in this cohort. Cronbachs alpha for BIS-11 total (0.83), attention (0.67), non-planning (0.73) and motor (0.56) scores in the Basel sample was in line with previous studies (McCarthy et al., 2016, Stanford et al., 2009). As done in previous studies (Pizzagalli et al., 2009, Pizzagalli et al., 2005, Schaub et al., 2021), a ‘anhedonic subscore’ for each patient was calculated with a total score on items from the Beck Depression Inventory II (BDI-II) (Beck et al., 1996) associated with anhedonic symptoms: loss of pleasure (item #4), loss of interest (item #12), loss of energy (item #15), and loss of interest in sex (item #21). Internal consistency of this subscore was acceptable (Cronbachs alpha α = 0.78).

### 2.3 MRI data acquisition

The Basel sample was scanned using a 3 T MRI system (Siemens Magnetom Prisma, Erlangen, Germany) and a 20-channel phased-array radio frequency head coil. Head movement was minimized by foam padding across the forehead. A whole brain 3-dimensional T1-weighted magnetization prepared rapid acquisition gradient (MPRAGE) sequence was applied. 176 slices were acquired in 4:08 min with a field of view of 256 mm^2^, voxels size 1 mm^3^ isotropic spatial resolution, inversion time of 1000 ms, repetition time of 2000 ms, echo time of 3.37 ms, flip angle of 8° and bandwidth of 200 Hz/pixel. The Zurich sample was scanned using a Philips Achieva 3 T whole-body scanner equipped with a 32-channel receive-only phased-array head coil (Philips Healthcare, Best, The Netherlands). Whole brain 3-dimensional T1-weighted anatomical data were obtained by using a MPRAGE with the following parameters: The MPRAGE sequence acquired 160 slices in 7:32 min with a field of view of 240 mm^2^, voxels size 1 mm^3^ isotropic spatial resolution, inversion time of 1008 ms, repetition time of 2987 ms, echo time of 3.7 ms, flip angle of 8° and bandwidth of 192 Hz/pixel. Raw images in both centres were assessed by trained neuroradiologists for radiological abnormalities.

### 2.4 Voxel-based morphometry (VBM)

MRI data were analysed with the standard automated processing stream of FSL-VBM (Douaud et al., 2007) (https://fsl.fmrib.ox.ac.uk/fsl/fslwiki/FSLVBM), an optimized VBM protocol (Good et al., 2001b) performed with FSL tools (Smith et al., 2004). The standard and optimized VBM protocol has been validated with highly reproducible segmentation results (Good et al., 2001a, Good et al., 2001b, Good et al., 2002, Voets et al., 2008). First, structural images were brain extracted and grey matter segmented before being registered to the 2 mm MNI 152 standard space using nonlinear registration. The resulting images were averaged and flipped along the x-axis to create a left–right symmetric, study-specific grey matter template. Second, all native grey matter images were nonlinearly registered to this study-specific template and “modulated” to correct for local expansion (or contraction) due to the nonlinear component of the spatial transformation. The modulated grey matter images were then smoothed with an isotropic Gaussian kernel with a sigma of 3 mm. The outputs of each VBM step were visually checked by authors (ACS, AS). In practice, all VBM steps did not require any manual interventions.

### 2.5 Statistical analyses

#### 2.5.1 Impulsivity

Analysis of covariance (ANCOVA) was conducted to examine group differences in BIS-11 total score and the BIS-11 2nd order factors attention, motor and non-planning, controlled for age, gender and smoking. Tukey post-hoc testing was further performed in case of significant F tests.

#### 2.5.2 Brain volume - symptom correlation analyses

A voxel-wise general linear model (GLM) was applied with nonparametric permutation (5000) tests (randomise (Nichols and Holmes, 2002)) using a single-group average design with additional covariates to test dimensional relationships between whole-brain grey matter volume and global impulsivity (BIS-11 total scores). Positive and negative associations between whole-brain grey matter and impulsivity scores were tested by controlling for age, gender (dummy variable), smoking (number of cigarettes per day), diagnosis (dummy variable), intracranial volume and scanner (dummy variable). Medication was also added as a categorical (dummy) variable, with ‘0′ for ‘no medication’, ‘1′ for ‘diacetylmorphine’, ‘2′ for ‘antidepressants’, ‘3′ for ‘antipsychotics’ and ‘4′ for ‘antidepressants + antipsychotics’. The statistical maps were thresholded at p < 0.05, family-wise error (FWE) corrected for multiple comparison using the threshold-free cluster enhancement (TFCE) technique (Smith and Nichols, 2009). The main analysis was conducted across the entire sample (n = 234). Although medication and scanner were included as covariates in this analysis, to further control for potential confounding effects, we conducted subanalyses in unmedicated (n = 178) and medicated (n = 56) patients and each centre separately. As such, these additional (sensitivity) analyses with subsamples were not corrected for multiple testing. Across HCs and patients with OUD and BPD with available BIS-11 2nd order factors (Basel sample (n = 116)), we conducted additional subanalyses to test the relationships between grey matter volume and the subfactors ‘attentional’, ‘motor’ and ‘non-planning’.

The same design was used to test dimensional relationships between BDI anhedonia scores and grey matter volume across all patients (n = 125). In line with our previous study (Schaub et al., 2021), this analysis was restricted to the bilateral nucleus accumbens, caudate and putamen. The Harvard-Oxford subcortical structural atlas as implemented in FSL was used to create one anatomical ROI mask (Supplementary Fig. 1).

In case of significant brain-symptom associations, FSLUtils (fslstats and fslmeants) was used to extract individual volumina (mm^3^) from significant clusters to depict the relationship in summary scatterplots. Spearman’s rho was further used to report the strengths of significant correlations.

#### 2.5.3 Behavioural and neural relationships between impulsivity and anhedonia

Exploratory testing of associations between BIS-11 impulsivity scores, anhedonia and their related volumetric brain correlates across patients were performed using partial correlation analyses in SPSS, controlled for group, age, gender, smoking, medication and centre (if data for both centres were available). Analyses with anhedonia were further controlled for depressive symptoms using the BDI total score without anhedonia items.

## 3 Results

### 3.1 Transdiagnostic impulsivity expression

**Fig. 1:**
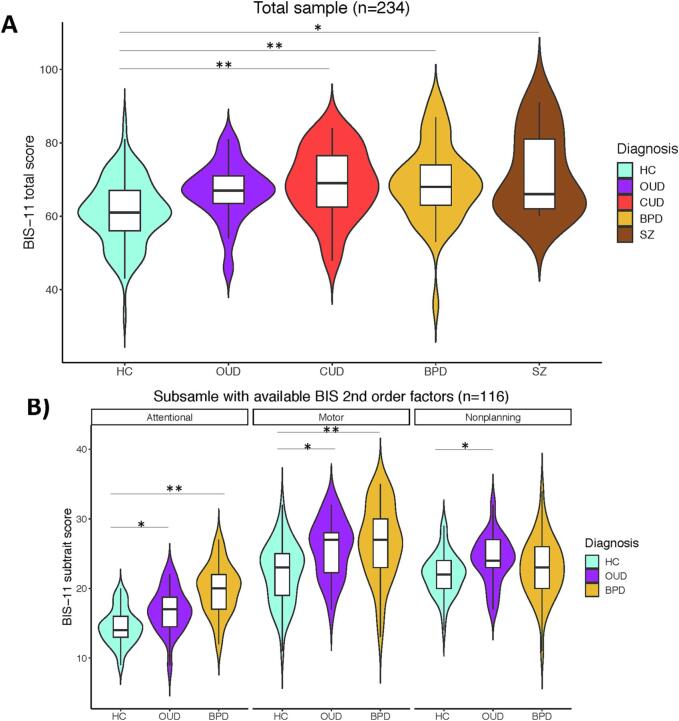
A) BIS-11 total scores for the entire multi-site sample consisting of 109 healthy controls (HCs), 22 patients with opioid use disorder (OUD), 43 patients with cocaine use disorder (CUD), 45 patients with borderline personality disorder (BPD) and 15 patients with schizophrenia (SZ). B) BIS-11 2nd order factors attentional, motor and non-planning across 49 HC, 22 OUD and 45 BPD patients (Basel sample, n = 116). * indicates statistically significant differences at p < 0.05 and ** indicates statistically significant differences at p < 0.001.

BIS-11 total scores were normally distributed across the entire sample (n = 234) (Shapiro-Wilk W = 0.992, p = 0.201). BIS-11 total scores differed between HCs and patients with CUD, OUD, SZ and BPD (F_4, 233_ = 8.415, p < 0.001) (Fig. 1A). Post-hoc testing showed significantly lower scores in HCs relative to SZ (p = 0.021), and BPD (p < 0.001), CUD (p < 0.001) and a trend compared with OUD patients (p = 0.088). None of the clinical groups significantly differed on the BIS-11 total score.

In the Basel sample (n = 116), BIS-11 total (Shapiro-Wilk W = 0.987, p = 0.322), attentional (Shapiro-Wilk W = 0.983, p = 0.163), motor (Shapiro-Wilk W = 0.985, p = 0.211) and non-planning (Shapiro-Wilk W = 0.987, p = 0.356) scores were also normally distributed. BIS-11 total scores significantly differed between HCs, OUD and BPD patients (F_2, 115_ = 14.058, p < 0.001), with higher scores in BPD (p < 0.001) and OUD patients (p = 0.002) relative to HCs (Fig. 1B). Regarding the 2nd order factors, significant group differences were evident for attention (F_2, 115_ = 27.918, p < 0.001), non-planning (F_2, 115_ = 8.407, p < 0.001) and motor impulsivity (F_2, 115_ = 3.287, p = 0.041). Post-hoc tests showed higher attentional and non-planning BIS-11 scores in BPD (p’s < 0.001) and OUD patients (p’s = 0.005) compared with HCs, and higher motor scores in OUD patients relative to HCs (p = 0.041).

### 3.2 Impulsivity maps onto the left inferior frontal gyrus across diagnoses

**Fig. 2:**
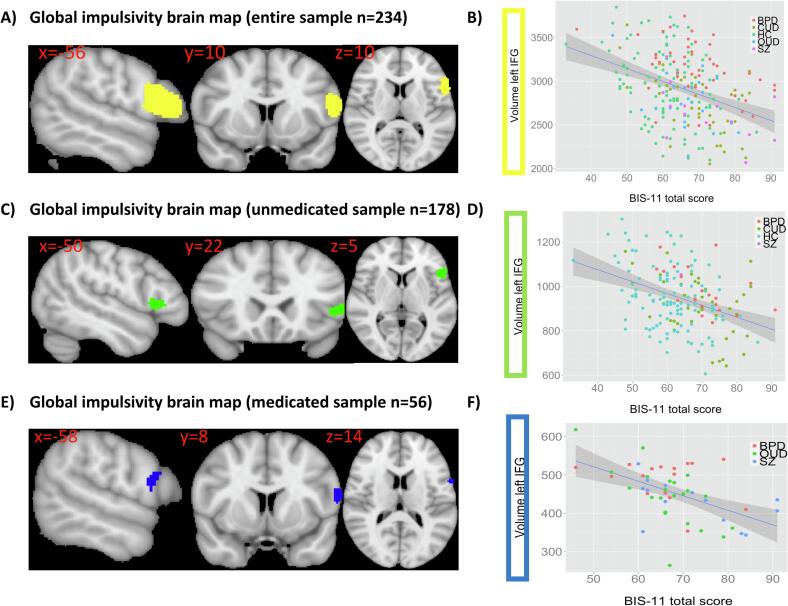
A) Significant negative relationship between global impulsivity (BIS-11 total scores) and volume in the left inferior frontal gyrus pars opercularis across the *entire multi-site sample* including HCs and patients with OUD, CUD, BPD and SZ (p_FWE_ < 0.001, cluster size = 753 voxels). Left hemisphere is displayed on the right. B) Summary scatterplot showing negative relationship between BIS-11 total scores and grey matter volume in the left inferior frontal gyrus (mm^3^) across the *entire multi-site sample* (r = -0.346). C) Negative association between BIS-11 total score and left inferior frontal gyrus volume across *unmedicated patients* (p_FWE_ = 0.015, cluster size = 215 voxels). D) Scatterplot depicting the negative relationship between BIS-11 total scores and volume in the left inferior frontal gyrus (mm^3^) in *unmedicated patients* (r = -0.340). E) Negative association between BIS-11 total score and left inferior frontal gyrus volume across *medicated patients* (p_FWE_ = 0.013, cluster size = 125 voxels). F) Scatterplot depicting the negative relationship between BIS-11 total scores and volume in the left inferior frontal gyrus (mm^3^) in *medicated patients* (r = -0.504).

**Fig. 3:**
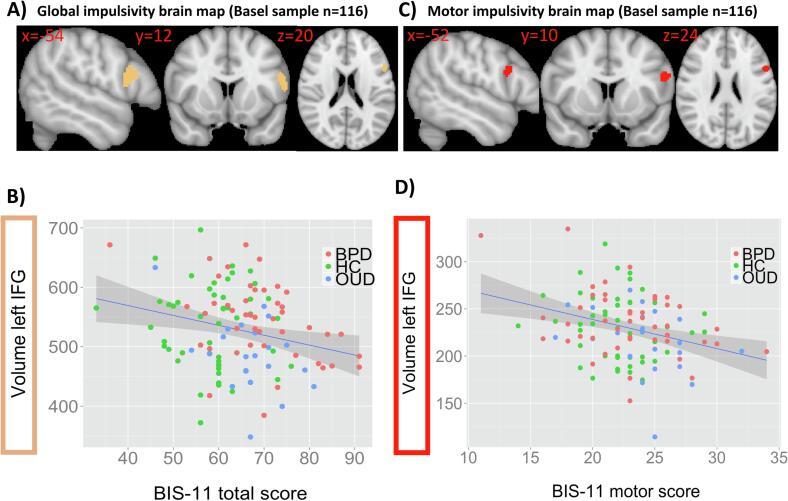
A) Validation of negative relationship between global impulsivity (BIS-11 total score) and inferior frontal gyrus volume across HCs and OUD and BPD patients (Basel sample, n = 116) (p_FWE_ = 0.003, cluster size = 113 voxels). Left hemisphere is displayed on the right. B) Summary scatterplot illustrating the negative association between left inferior frontal gyrus volume (mm^3^) and BIS-11 total scores across HCs and OUD and BPD patients (r = -0.232). C) Brain map showing the negative association between BIS-11 motor scores and inferior frontal gyrus volume across HCs and OUD and BPD patients (p_FWE_ = 0.022, cluster size = 51 voxels). D) Scatterplot for the negative relationship between BIS-11 motor scores and volume in the left inferior frontal gyrus (mm^3^) across HCs and OUD and BPD patients (r = -0.254).

Across the entire sample, there was a significant negative relationship between BIS-11 total scores (global impulsivity) and volume in the left IFG (pars opercularis) (Fig. 2A). This association was moderate (r = -0.346) as shown in the summary scatterplot (Fig. 2B). The same relationship was also evident across all patients (Supplementary Fig. 2), as well as in unmedicated (Fig. 2C and D) and medicated patients separately (Fig. 2E and F). Site-specific analyses confirmed the negative relationship between left IFG volume and global impulsivity, in both the Basel (Fig. 3A and B) and Zurich sample (n = 118, Supplementary Fig. 3).

Subanalyses with available BIS-11 2nd order factors across HCs, BPD and OUD patients from the Basel sample further revealed a negative relationship between left IFG volume and the BIS-11 motor factor (Fig. 3C) but not with the attention and non-planning factor. As shown in the summary scatterplot this correlation was moderate (r = -0.254) (Fig. 3D).

### 3.3 Anhedonia is related to left putamen volume across diagnoses

Across BPD, CUD, OUD and SZ patients (n = 125), BDI anhedonia scores were negatively related to the volume of the left putamen (p_FWE_ = 0.029, cluster size = 52 voxels, Supplementary Fig. 4).

### 3.4 Associations between impulsivity and anhedonia and their neural substrates

**Fig. 4:**
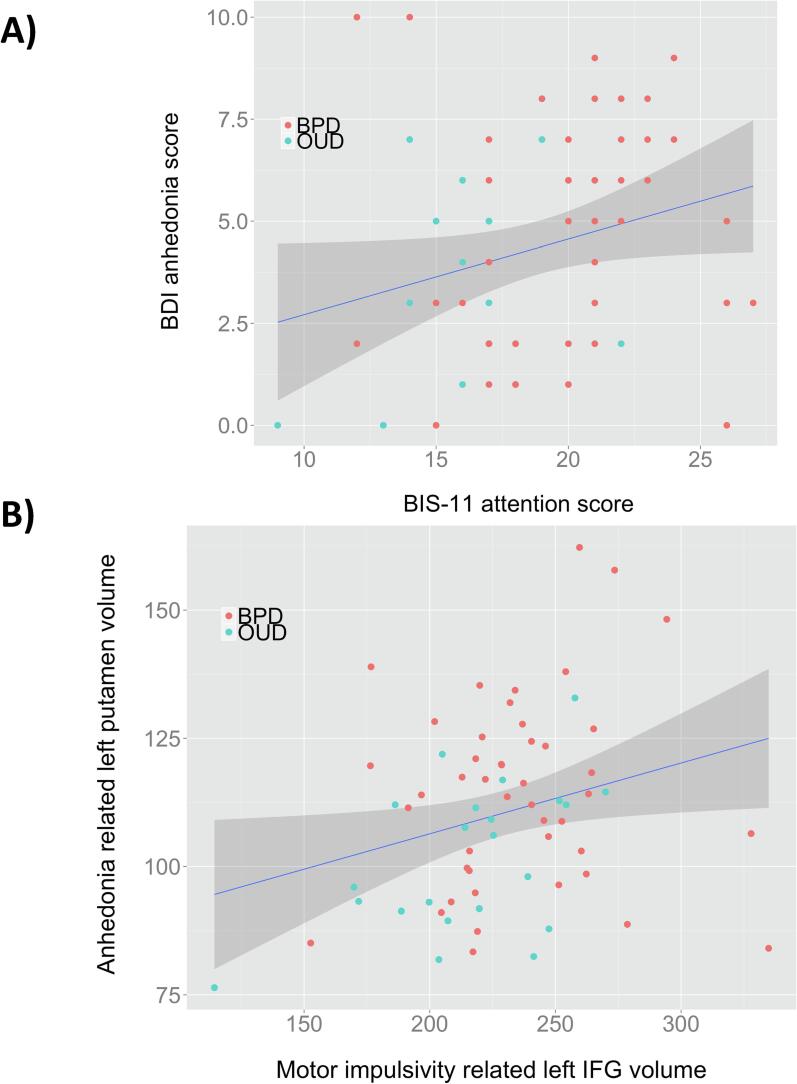
A) Scatterplot depicting the positive association between BIS-11 attention scores and BDI anhedonia scores across OUD and BPD patients (n = 67, r = 0.305, p = 0.012). B) Significant positive relationship between anhedonia related volume in the left putamen (mm^3^) and motor impulsivity related volume in the left IFG across OUD and BPD patients (r = 0.267, p = 0.029).

Across all patients, there was no relationship between global impulsivity and BDI anhedonia scores (r = 0.101, p = 0.263), as well as between their neural substrates, the left IFG and left putamen (r = -0.043, p = 0.632). However, across OUD and BPD patients in the Basel cohort with available BIS-11 2nd order factors, we found that anhedonia scores were positively related to BIS-11 attentional scores (r = 0.305, p = 0.012) (Fig. 4A) but not to total (r = 0.100, p = 0.419), motor (r = -0.032, p = 0.797) or non-planning scores (r = -0.039, p = 0.753). Furthermore, anhedonia-associated left putamen volume was positively associated with motor impulsivity associated left IFG volume across OUD and BPD patients (r = 0.267, p = 0.029) (Fig. 4B).

## 4 Discussion

The main result of this ad hoc study is that global impulsivity mapped onto the volume of the left IFG pars opercularis across healthy participants and patients with OUD, CUD, BPD and SZ. Subsequent analysis in a sample of HC, OUD and BPD patients further indicates that the left IFG volume might be specifically related to motor impulsivity. Secondary and more exploratory findings across OUD and BPD patients suggest that attentional impulsivity was positively associated with anhedonia expression, whereas motor impulsivity associated volume in the left IFG was positively correlated with anhedonia-associated volume in the left putamen.

### 4.1 Associations between impulsivity and left IFG volume

In line with our main result of a negative association between BIS-11 total scores and left IFG volume across healthy individuals and patients with OUD, CUD, BPD and SZ, previous evidence from voxel-based lesion-symptom mapping in patients with traumatic brain injuries showed that global impulsivity (BIS-11 total scores) is associated with damage to the left IFG (McDonald et al., 2017). Compared to subjects with low BIS-11 total scores, healthy participants with high BIS-11 total scores also exhibited significant cortical thinning in the left IFG pars opercularis, which correlated negatively with BIS-11 total scores (Lim et al., 2021). This fits with another study in healthy individuals showing that higher BIS-11 total scores were associated with a thinner cortex in the left IFG (Tu et al., 2017). Furthermore, reduced myelination in the left IFG over one year was related to increased BIS-11 total scores in healthy participants (Ziegler et al., 2019). In adults with attention deficit hyperactivity disorder (ADHD), impulsivity (Adult ADHD Self-Rating Scale) was also negatively correlated with volume in the left IFG (Klein et al., 2021), whereas between centrality of the left IFG was negatively related to BIS-11 total scores in patients with conduct disorder (Lu et al., 2017). In BPD patients, BIS-11 total scores correlated negatively with left IFG activation during attention allocation (Wrege et al., 2022).

In a subsample of healthy participants, OUD and BPD patients with available BIS-11 2nd order factors, we further found a more specific negative relationship between left IFG volume and BIS-11 motor impulsivity. Although more emphasis has been put on the role of right IFG in response inhibition (Aron et al., 2004, Aron et al., 2014), our finding is in accordance with previous studies showing a relationship between the left IFG and response inhibition, i.e. motor impulsivity (Goya-Maldonado et al., 2010, McDonald et al., 2017, Swick et al., 2008, Swick and Chatham, 2014, Wager et al., 2005). However, it is of note that performance on response inhibition tasks is not always related to self-reported impulsivity (Eisenberg et al., 2019, Sánchez-Kuhn et al., 2017). Another point of contention is that the three-factor structure (i.e. motor, attention, non-planning) of the BIS-11 is questionable (Reise et al., 2013, Steinberg et al., 2013). In our study internal consistency of the BIS-11 motor factor (Cronbach’s α 0.56) did also not meet reliability acceptability, hence the specific relationship between BIS-11 motor impulsivity scores and left IFG volume needs to be interpreted cautiously. The non-specificity of the left IFG volume to the BIS-11 motor factor is also reflected in other studies showing negative relationships between left IFG volume and BIS-11 attentional and non-planning in patients with CUD (Meade et al., 2020) and alcohol use disorder (AUD) (Gröpper et al., 2016). Instead of mediating response inhibition/motor impulsivity, whereby the right IFG is considered as key node (Aron et al., 2004, Aron et al., 2014, Schroeder et al., 2022), the left part of IFG may rather be involved in mediating attentional processing of the stop signal (Rubia et al., 2001, Swick et al., 2008) or conflict resolution (Grinband et al., 2011, Nee et al., 2007, Roberts and Hall, 2008, Roelofs et al., 2006), specifically in overriding highly regularized, automatic processes (Novick et al., 2005). In particular it has been proposed that the left IFG affects the processing of observed actions through descending inhibitory processes and that attentional modulation of the left IFG is responsible for filtering task-irrelevant actions during ongoing behaviour (Chong et al., 2008). This is supported by a previous study showing that the left IFG is involved in adjusting response bias with respect to the context and thus enabling flexible decision-making (Reckless et al., 2014) and by the selection hypothesis, which considers left IFG as a general mechanism for selecting among competing representations (Thompson-Schill, 2003, Zhang et al., 2004). In accordance, increased volume and cortical thickness of the left IFG over time were associated with improved cognitive flexibility, decision-making (Parvaz et al., 2017) and sustained attention (Hirsiger et al., 2019) in CUD patients. Considering this evidence, our findings of left IFG-BIS-11 (total and motor scores) associations may reflect a more general deficits in attentional control mechanisms that occur across psychiatric diagnoses.

### 4.2 Behavioral association between anhedonia and impulsivity

We further found that anhedonia was positively related to attentional impulsivity (inability to focus on current tasks and intruding thoughts) across HC, OUD and BPD patients (subsample with additional data on BIS-11 2nd order factors). High anhedonia has previously been related to low attentional control in healthy adults (Tully et al., 2014) and a recent network analysis in healthy participants showed that trait anhedonia was connected to BIS-11 non-planning and attention (Zhang et al., 2022). A positive association between BIS-11 attentional impulsivity and anhedonia has already been reported in patients with bipolar disorder (Swann et al., 2008) and anhedonia was also found to be positively related to dysfunctional impulsivity (acting with less forethought) in BPD patients (Marissen et al., 2012). A previous study in nonclinical anhedonic subjects further revealed deficits in sustained attention as expressed by smaller P300 event-related potentials (Dubal et al., 2000). Attentional control is critical for the management of both positive and negative affect (Vasey et al., 2013). Deficits in attentional control may impair down-regulation of negative affect and up-regulation of positive affect, which is proposed to underlie anticipatory anhedonia (Pizzagalli, 2010). In the same vein, anticipatory pleasure deficits have been associated with the inability to encode the reward value of future pleasurable activities, a process involving attentional control functions (Burbridge and Barch, 2007). Therefore, our finding of positive anhedonia – attentional impulsivity relationship may suggest that impaired attentional control mechanisms in OUD and BPD patients result in an inability to generate future reward representations and in turn in a lack of motivation to engage in pleasurable activities.

### 4.3 Neural association between anhedonia and impulsivity

As previously shown in the same sample as used in the present study including patients with depression and first-episode psychosis (Schaub et al., 2021), we found that anhedonia expression was negatively related to left putamen volume across patients with BPD, CUD, OUD and SZ. This finding suggests that the previously found negative anhedonia-putamen association was not driven by depressed patients. Based on its involvement in the acquisition of stimulus-action-reward associations (Haruno and Kawato, 2006) and evidence showing that inhibitory dysfunction of the putamen in monkeys reduced the frequency of self-initiated actions to collect reward (Worbe et al., 2009), the reduced putamen volume might reflect psychomotor retardation or diminished motor drive to initiate approach behaviour. Intriguingly, here we further found that the anhedonia-associated left putamen volume correlated with motor impulsivity-associated left IFG volume across BPD and OUD patients. In a transdiagnostic sample of young adults, a recent study showed a positive relationship between negative urgency, as measured with the UPPS-P Impulsive Behavior Scale (UPPS-P, Whiteside and Lynam, 2001), and left ventrolateral PFC activity during reward expectancy (Edmiston et al., 2020), indicating that reward expectancy-related left ventrolateral PFC may represent a state of frustration or impatience during reward anticipation. This finding further underpins the role of the left ventrolateral PFC in impulsivity and reward sensitivity (Joseph et al., 2009, Krebs et al., 2009) and approach behavior (Davidson et al., 2004). Moreover, uncertain reward expectancy-related activity in the left ventrolateral PFC was associated with high trait impulsive sensation seeking in young adults (Chase et al., 2017). As the left IFG, the putamen has also been implicated both in motor impulsivity (Chambers et al., 2009) and motivation (Schultz, 2000). Its involvement is critical for the interaction of cognitive inhibitory operations and motivational processes (Padmala and Pessoa, 2010). In CUD patients, lack of premeditation and UPPS-P negative urgency (the tendency to engage in rash, ill-considered action in response to intense negative emotions) was associated with reduced volumes in both left putamen and left IFG (Moreno-López et al., 2012) and altered putaminal white matter integrity was associated with heightened impulsivity in current and past methamphetamine users (Andres et al., 2016). Furthermore, decreased myelination of the ventral putamen has been associated with motor impulsivity in a serial reaction time task in youth (Nord et al., 2019). Both volumetric changes in the dorsal striatum and inferior prefrontal cortex have been associated with increased risk for developing stimulant drug dependence (Ersche et al., 2012). Our result is thus in line with a body of evidence indicating the involvement of the putamen in impulsive behaviour and reward seeking in neuropsychiatric disorders (Luo et al., 2019).

### 4.4 Limitations

Our interpretations should be taken in the context of possible limitations. This was an ad hoc transdiagnostic MRI investigation using available self-report data of trait impulsivity and anhedonia. While the association between global impulsivity and left IFG was observed in a large sample and with a BIS-11 scale (total score) with good internal consistency (Cronbach’s α = 0.83), the differentiation between impulsivity subfactors and related brain regions was only possible in a subsample with modest size (n = 116). Further, we were only able to test for these associations in OUD and BPD patients, questioning whether they were also evident across other diagnoses. This also holds for the main analysis, where only patients with OUD, CUD, BPD and SZ were included. The generalizability of the main finding for SZ is also limited given that only 15 patients with this disorder could be included. Furthermore and in line with previous studies (McCarthy et al., 2016, Stanford et al., 2009), internal consistency of the BIS-11 attentional and motor factors (Cronbachs α 0.67 and 0.56, respectively) did not meet reliability acceptability and therefore the results of these subanalyses should be interpreted with caution. Although the BIS-11 has been influential in psychiatric impulsivity research, the UPPS-P (Whiteside and Lynam, 2001) and the Three-Factor Impulsivity Index (Johnson et al., 2017) are better constructed to identify transdiagnostic neural correlates of emotion-related impulsivity (Johnson et al., 2020). With respect to laterality and emotion-related impulsivity, it is of note that a recent study found a negative relationship between cortical gyrification in the right lateral OFC and high emotion-related impulsivity as expressed by the Three-Factor Impulsivity Index in a transdiagnostic sample (Elliott et al., 2023). In the same vein as with the BIS-11, a rather broad measure of anhedonia was used in the current post-hoc study as expressed by the BDI anhedonia subscale, which do not allow a differentiation between the consummatory and anticipatory aspect of anhedonia. Finally, although we controlled our analyses for the different types of medication and were able to show the same findings in unmedicated and medicated individuals, we cannot directly infer on medication effects. Bearing in mind the critical involvement of dopamine in both impulsivity and anhedonia (Buckholtz et al., 2010, Husain and Roiser, 2018), it would be enlightening to conduct an exploration in patients before and after dopaminergic treatment.

## 5 Conclusions

In conclusion, this study provides solid evidence for a negative association between grey matter volume of the left IFG and global impulsivity across a transdiagnostic sample of healthy individuals and patients with OUD, CUD, BPD and SZ, which might reflect a general impairment in attentional control and conflict resolution mechanisms. Further preliminary results in OUD and BPD patients suggest associations between impulsivity subscales and anhedonia that may involve concomitant volume reduction in the left putamen and IFG. Future a priori defined works should validate our findings in large transdiagnostic samples and further investigate association with impulsivity and anhedonia with more specific assessments.

## CRediT authorship contribution statement

**Anna-Chiara Schaub:** Data curation, Formal analysis, Writing – original draft, Writing – review & editing. **Marc Vogel:** Writing – review & editing. **Undine E. Lang:** Writing – review & editing. **Stefan Kaiser:** Writing – review & editing. **Marc Walter:** Writing – review & editing. **Marcus Herdener:** Writing – review & editing. **Johannes Wrege:** Writing – review & editing. **Matthias Kirschner:** Data curation, Writing – review & editing. **André Schmidt:** Conceptualization, Formal analysis, Funding acquisition, Methodology, Project administration, Resources, Supervision, Validation, Writing – original draft, Writing – review & editing.

## Declaration of Competing Interest

The authors declare that they have no known competing financial interests or personal relationships that could have appeared to influence the work reported in this paper.

## Data Availability

Data will be made available on request.
