# Supplementary material for: Transdiagnostic brain correlates of self-reported trait impulsivity: A dimensional structure-symptom investigation
Source: Neuroimage Clin. 2023 Apr 28;38:103423. doi: 10.1016/j.nicl.2023.103423 (PMC10176059; doi:10.1016/j.nicl.2023.103423)
Supplement: Supplementary data 1 [file mmc1.docx]

**Transdiagnostic brain correlates of self-reported trait impulsivity: a dimensional structure-symptom investigation**

Supplementary information

**Supplementary Figure 1.** Region of interest mask for the brain volume – anhedonia analysis. **Dorsal striatum:** bilateral caudate (yellow) and bilateral putamen (blue); **Ventral striatum**: bilateral nucleus accumbens (green). The Harvard-Oxford subcortical structural atlas as implemented in FSL was used to create the ROI mask.

**
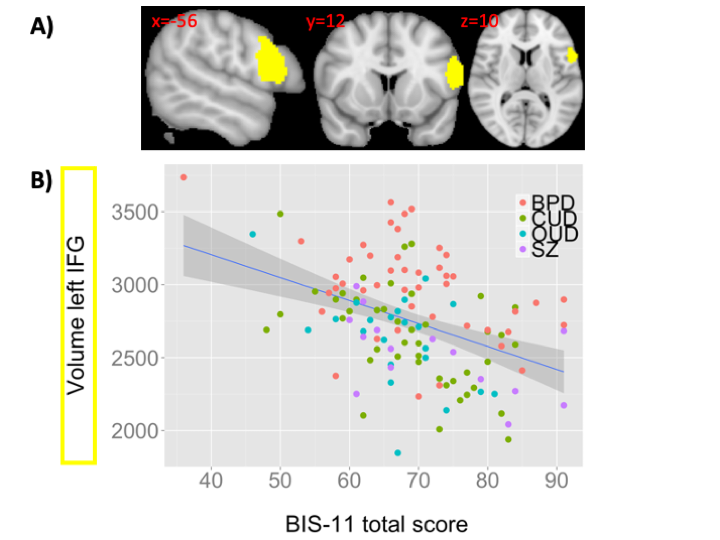
**

**Supplementary Figure 2. A)** Significant negative relationship between BIS-11 total scores and volume in the left inferior frontal gyrus (pars opercularis) *across all patients* including diagnoses of borderline personality disorder (BPD), cocaine use disorder (CUD), opioid use disorder (OUD) and schizophrenia (SZ) (p_FWE_<0.001, cluster size=733 voxels). Left hemisphere is displayed on the right. **B)** Summary scatterplot depicting the negative relationship between BIS-11 total scores and grey matter volume in the left inferior frontal gyrus across all patients (r=-0.418).


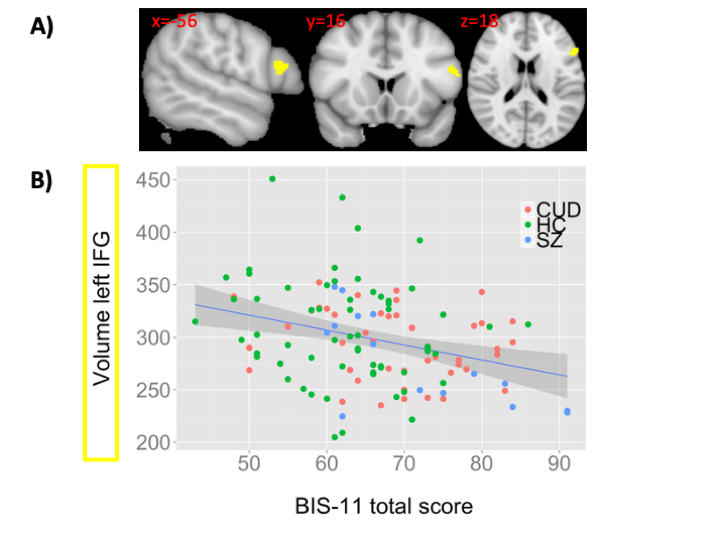


**Supplementary Figure 3. A)** Significant negative relationship between BIS-11 total scores and volume in the left inferior frontal gyrus in the *Zurich sample* including patients with cocaine use disorder (CUD), schizophrenia (SZ) and healthy controls (HC) (p_FWE_=0.013, cluster size=82 voxels). Left hemisphere is displayed on the right. **B)** Scatterplot showing negative relation between BIS-11 total scores and grey matter volume in the left inferior frontal gyrus in the Zurich sample (r=-0.329).

**
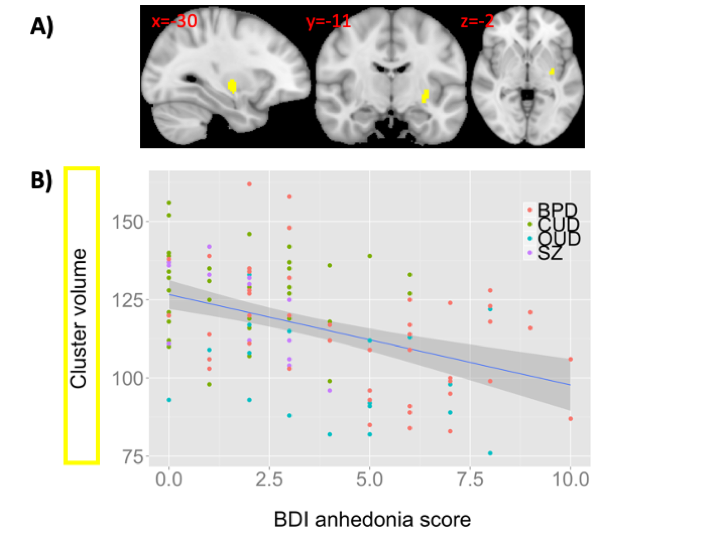
**

**Supplementary Figure 4. A)** Significant negative relationship between BDI anhedonia scores and volume in the left putamen across all patients (p_FWE_=0.029, cluster size=52 voxels). Left hemisphere is displayed on the right. **B)** Scatterplot showing negative relation between BDI anhedonia scores and grey matter volume in the left putamen across all patients (r=-0.338).
